# Clinical application of low-level laser therapy (Photo-biomodulation therapy) in the management of breast cancer-related lymphedema: a systematic review

**DOI:** 10.1186/s12885-022-10021-8

**Published:** 2022-08-30

**Authors:** Dania Mahmood, Ashfaq Ahmad, Faiza Sharif, Syed Asadullah Arslan

**Affiliations:** University Institute of Physical Therapy, Faculty of Allied Health Sciences, The University of Lahore, Lahore, Pakistan

**Keywords:** Low-level laser therapy, Photo-biomodulation therapy, Laser therapy, Breast cancer, Lymphedema, Postmastectomy lymphedema, Systematic review

## Abstract

**Background:**

Breast cancer-related lymphedema (BCRL) is a frequent issue that arises after mastectomy surgery in women and compromises physical and mental function. Previously published studies have shown positive effects with the use of Low-level laser therapy in another term Photo-biomodulation therapy (PBM). This research investigated the efficacy of clinical use of LLLT (PBM) in the treatment of metastatic breast cancer-related lymphedema.

**Methods:**

PubMed, PEDro, Medline, and the Cochrane Library were searched for LLLT clinical trials published before October 2021. The methodological quality of randomized trials and the effectiveness of Laser Therapy for BCRL were evaluated. The primary objectives were arm circumference or arm volume, whereas the secondary goals were to assess shoulder mobility and pain severity.

**Results:**

Eight clinical trials were analyzed in total. Typically, the included RCTs had good research quality. At four weeks, there was a considerable reduction in arm circumference/volume, and this continued with long-term follow-up. However, no statistically significant change in shoulder mobility or pain severity was seen between the laser and placebo groups at 0-, 1-, 2-, and 3-month short-term follow-up.

**Conclusions:**

The findings of this comprehensive study demonstrated that LLLT (PBM) was successful in diminishing arm circumference and volume than improving shoulder mobility and pain. Data indicates that laser therapy (PBM) may be a beneficial treatment option for females with PML. Because of the scarcity of evidence, there is a strong need for well-conducted and longer-duration trials in this field.

**Trial registration:**

Details of the protocol for this systematic review were registered on PROSPERO and can be accessed at www.crd.york.ac.uk/PROSPERO/display_record.asp?ID=CRD42022315076.

**Supplementary Information:**

The online version contains supplementary material available at 10.1186/s12885-022-10021-8.

## Background

Breast cancer is still frequent cancer in women all over the world. An aberrant expansion of cells in the breast tissue is what it is called. Milk-producing glands called lobules and ducts that link the lobules to the nipple make up the tissues. Lymphatic, connective, and fatty tissues make up the remainder of the breast. The carcinoma spreads to the lining cells (epithelial tissue) of the glandular tissue's ducts or lobules. In industrialized nations (e.g., Europe, the United States, and Japan), about 82 percent of women survive ten years after being diagnosed with breast cancer [1]. Although Asian countries have a lower incidence of breast cancer, cause-specific mortality is substantially greater in most Asian countries than in Western ones [2].

Lumpectomy (removal of the tumor) or Mastectomy (surgical removal of the entire breast) are well-known treatment options for breast cancer. These are continuing operations for woman’s survival from breast cancer, depending on the spread stage. In general, women are under-informed about the condition and its potential repercussions. Lymphedema has always been the most prevalent problem following therapy. *Lymphedema* is a chronic disorder in which protein-rich edema accumulates in the tissue spaces [3]. Dysfunction in the axillary drainage system induced by surgeries or laser therapy causes it to worsen. All lymph fluid drains to the axillary lymph nodes from one side of the upper body (chest, ribcage, arm, and hand). This flow is more prone to be affected when more lymph nodules and veins are removed, and could result in lymphoedema.

Women experience a variety of issues related to breast cancer therapy, aside from edema buildup in the affected arm. Patients experiences some medical conditions as a result of side effects after therapy. Cancer treatments are effective in killing cancer cells, but most of them also harm healthy cells and can alter a woman's physical or emotional state. During heavy doses of chemotherapy, radiation therapy, and hormone therapy, a woman may undergo symptoms such as poor appetite, nausea, vomiting, weakness, and hair loss. Physical appearance and psychological thoughts are both impacted by external body changes, which can cause depression, sadness, and a sense of loneliness. Exercise is one of the best strategies for managing these conditions. When used for longer than three months, a rehabilitation programme that incorporates yoga and various forms of exercise has shown to control mood swings in women [4].

As a result of long-term side effect of treatment, lymphedema causes swelling in the limbs, persistent inflammation, tissue tearing, infection, and limited motion. In addition, swelling, heaviness, hardness, tenderness, soreness, numbness, itching, and stiffness are among the signs of lymphedema in breast cancer survivors [5, 6]. Although there are many different methods for measuring arm volumes, including traditional volumetry with overflow, volumetry without overflow, and inverse volumetry, but volume based on arm circumference is still the most common one used [7]. Even though standard approach is still the best option for measuring arm fluid, a new portable three-dimension laser system (called 3DLS) for measuring upper limb volume has also produced promising findings for the diagnosis of lymphedema [8]. To be more specific, the 3DLS technology uses a triangulation process that involves projecting a laser dot onto an object (in this case, the upper limb) to represent the 3D model, and then a sensor calculates the distance to the item's surface. The 3DLS method for rapid volume measurement is a new standardized augmented reality-based technique [9].

There is no definitive medical or surgical cure for lymphedema, the fluid return is thought to be managed by standard therapy and physical activity. Surprisingly, a complete decongestive therapy (CDT) treatment can be utilized to reduce lymphedema rates. Multilayer bandaging (MLB), compression therapy, bilateral lymphatic drainage, and a healthy exercise regimen are all part of the treatment. Furthermore, secondary lymphedema can be treated conservatively without harm from the given interventions [10]. Nowadays, laser therapy has been utilized to treat (PML) postmastectomy lymphedema. Although it has been in use since past 2 decade,but due to its high level of demand it is being clinically being applied for various medical conditions. Moreover, laser treatment, also referred to as Low-level Laser Therapy (LLLT), has been demonstrated to help slow the progression of recurrent lymphedema caused by breast cancer.

Low-level laser therapy is a nonionizing light-based conservative therapy that has been utilized to treat lymphedema in women with breast cancer [11]. Photons of a specified wavelength (650 nm and 1000 nm) penetrate skin tissue to give low rays and doses to the targeted area in laser treatment or photo-biomodulation therapy (PBM). It has been implemented to help with lymphatic fluidity, redness, lymph vessel restoration, and tissue stiffness prevention [12–15]. Biochemical changes at the cellular level, on the other hand, are the critical mechanism for employing LLLT (PBM).

Fibroblasts, osteoblasts, lymphocytes, and smooth cells are all altered during the therapy. These effects result from instantaneous reactions involving the absorption of specific light wavelengths. The cytochromes, cytochrome oxidase, and flavin dehydrogenases in the mitochondrial respiratory chain absorb the rays, causing changes in the reduction–oxidation reaction (REDOX) state of the cytoplasm and mitochondria, which leads to increasing the levels of adenosine triphosphate (ATP) [16]. After (ATP) synthesis, an increase in metabolic energy triggers a subsequent critical process for cellular repair. Furthermore, intracellular signalling and cytokine activation allow for various responses, including the development of new lymphatic vessels, the release of growth factors, and metabolic upregulation [17–19]. As a result, LLLT (PBM) helps enhance the immune system by facilitating the drainage of excess protein-rich fluid and increasing macrophage formation.

The usefulness of laser for the therapy of breast cancer-related lymphedema has been the subject of little published research during the last twenty years (BCRL). The most current Systematic review, published in 2017, included Randomized Control Trials (RCTs) and observational studies with a follow-up of fewer than six months conducted between 1998 and 2013, revealing the RCTs' short-term follow-up [20]. Efficacy in treating women with BCRL has been studied in several RCTs. Five short studies of appropriate methodological quality were used by Omar et al. to provide moderate to strong evidence for the benefit of laser therapy in the treatment of BCRL [21]. Another clinical investigation, done by Carati CJ et al., had two experimental groups and reported that after two cycles of LLLT, the mean impacted limb volume tended to decrease over time [22].

As a result, the goal of this study was to gather all updated clinical studies published between 2010 and 2022. In addition, this study looked at clinical trials that focused on the efficacy of LLLT (PBM) for mature females with postmastectomy lymphedema with a follow-up of 6 months or more, intending to do research on the long-term effects of laser treatment based on the literature available. Furthermore, this study analysed the findings of recently published RCTs after 2017 for the use of laser treatment for BCRL. Finally, we undertook an updated assessment of all current LLLT (PBM) evidence for BCRL to address these difficulties.

## Methods

This study was performed under the guidelines by Preferred Reporting Items for Systematic Review and Meta-Analyses (PRISMA) [23, 24] statements.

### Literature search

The research review was restricted to studies published in English between 2010 and 2022. The relevant studies were found using four databases, including PubMed, Physiotherapy Evidence Database PEDro, Medline, and Cochrane Library. The keywords (photo-biomodulation) AND (lymphedema OR lymphoedema OR edema OR edoema OR swelling) AND (low-level laser therapy OR cold laser OR cold therapy OR low energy laser OR laser therapy) AND (breast cancer) were used to search the databases, with minor modifications for specific database queries. PICO criteria was used to answer appropriate clinical questions. Further research was discovered by looking through the reference sections of all pertinent articles. If necessary, experts were called in to identify things.

### Eligibility criteria

PICO criteria was used to illustrate the following criteria [25].

### Inclusion criteria

If an article met the following requirements, it was considered eligible.

1. **Types of study designs:** The researchers aimed for randomized controlled trials. (Single or double-blinded)
2. **Types of participants:** Adult women older than 18 years old having unilateral lymphedema secondary to Mastectomy or radiotherapy. The circumferential limb difference was defined as more than 2 cm up to 8 cm compared with contralateral (unaffected limb) to be classified as lymphedema.
3. **Type of Intervention applied:** Low-level laser therapy/ Photo-biomodulation treatment as a single therapy or combined therapy included. The control subjects had no restrictions, including no treatment, placebo laser, or combination therapy as an active treatment other than LLLT (PBM).
4. **Comparison:** There was no comparison to low-level laser therapy.
5. **Type of outcomes measure:** Arm circumference/volume, pain intensity, shoulder mobility, and subjective symptoms are clinically linked outcome factors.

### Exclusion criteria

The research excluded subjects with primary lymphedema or lymphedema caused by any disease other than breast cancer. Patients with bilateral lymphedema, active malignancy, pregnancy, or any cardiovascular disease or persistent inflammation were also excluded from the trial. Observational evaluations, suggestions, polls, remarks, columns, and emails were also excluded.

#### Study selection

Two reviewers first gathered the published paper by screening the titles and abstracts for their qualifying criteria. After then, the entire text was screened for final inclusion. Authors, institutions, publication journals, and study outcomes were not hidden from reviewers. The two reviewers thoroughly reviewed and graded each paper, and any disagreements between them were resolved through conversation.

#### Data extraction

Authors, publication year, study population and patients, treatment, co-intervention, outcomes, measurement period, findings, and inclusion criteria, and degree of evidence (if applicable) were all recorded by the reviewers using a standardized spreadsheet using Microsoft Excel for RCTs. The agreement was achieved through discussion. For further information (if needed), their respected authors were contacted.

#### Methodological quality appraisal

Each included trial's methodological quality was assessed using Physiotherapy Evidence Database (PEDro) scale, based on the Delphi list [26]. The PEDro scale comprises 11 components which include (eligibility criteria, random allocation, concealed allocation, baseline group comparison, blinding of the subject, blinding of the therapist, blinding of assessors, measuring time protocol, intention to treat analysis, comparison among groups and point estimate and variability). The Pedro points go from 1 to 10; the greater the PEDro score, the higher the study's quality. Because there was no established cut-off number for identifying a high-quality study, the following technique was used: a PEDro score of less than five indicated low-quality evidence, while a score of more than five indicated high-quality evidence [27, 28]. However, the scale's dependability was previously assessed and found to be acceptable (ICC00.68) by evaluators [29]. Additionally, two additional reviewers independently utilized the Cochrane Risk of Bias (2.0) tool [30] to evaluate the risk of bias for the included studies. Each domain's bias risk was categorized as high, low, or uncertain. Any dispute among two reviewers on overall rating of quality was settled with the assistance of third reviewer.

#### Outcomes assessments

The primary outcomes were based on the difference in the subjects' arm circumference or volume relative to the unaffected arm. Pain relief and an increase in range of motion were marked as secondary outcomes resulting from the use of LLLT (PBM). The results of the included RCTs were used in the primary analysis. The following criteria were used to separate the RCT data into control groups, outcome measures, and follow-up:

1. Comparison: Compression garment, manual lymphatic drainage (MLD), total decongestive therapy, and arm exercises were included in the conventional therapy group.
2. Outcome Measure: The primary objectives were arm volume and arm circumference, with the secondary endpoints being pain severity and shoulder mobility in the affected arm.
3. Measure time point: According to the accessibility of research, more than six months of long-term follow-up were included immediately after the conclusion of treatment sessions and 1- to 6 months of short-term follow-up were also considered.

Due to the small number of studies and clinical heterogeneity, meta-analysis was not achievable. However, to obtain the final conclusions, a high degree of evidence was considered, including methodological quality, original article results, and RCTs that revealed consistent findings.

##### Strong

Multiple high-quality RCTs have produced consistent results.

##### Moderate

Several low-quality and/or one high-quality RCT findings that are consistent.

##### Limited

A single poor-quality RCT.

##### Conflicting

Multiple RCTs produced conflicting results.

There is no proof from trials—no RCTs. [31].

### Review criteria

The articles were arranged as stated by Sackett’s evidence rule [32].

Levels of evidence from Sackett’s are as follows:

- Level 1, large RCTs with precise cut results.
- Level 2, small RCTs with unclear results.
- Level 3, non-randomized cohort, and case–control studies.
- Level 4, non-randomized historical cohort, or case–control studies.
- Level 5, case series without control groups.

Sackett’s level of evidence was used to rate the level of evidence to make the results more authentic.

### Assessment of laser therapy treatment

Laser therapy parameters (wavelength, laser model, energy density, output power and application zone) of included RCTs were used to analyse the treatment adequacy. These parameters were acknowledged as given by World Association for Laser Therapy (WALT) guidelines [33]. Reviewers who had vast knowledge of laser therapy assessed the PBM adequacy of the treatment protocol, and any disagreements were resolved by discussion.

This study was registered with PROSPERO (number CRD42022315076).

## Results

### Study selection

**Fig. 1:**
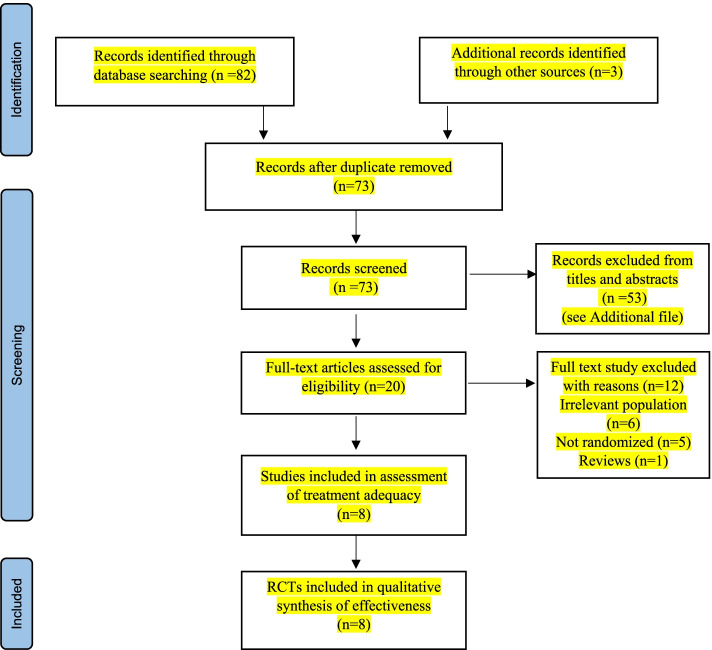
Flowchart of the selected clinical trials

Figure 1 depicts the review procedure. Through computerized and manual searches, the initial search yielded a total of 85 studies. After screening for duplicates, 73 records were considered valid, and after screening for titles and abstracts, 53 studies were eliminated. After considering the complete content of the remaining reports, we decided to eliminate further six of them from our final analysis. As a result, eight trials [21, 34–40] were chosen to be included in this review to see how effective Laser therapy (PBM) is for postmastectomy patients with unilateral lymphedema.

### Participants baseline characteristics

The baseline variables for the clinical studies that are included are shown in Table 1 Data of participants baseline characteristics of both Intervention group and Control group. Some studies do not indicate any baseline characteristics; hence they have been labelled as not reported. Out of many variables, the most prevalent ones have been reported.

**Table 1:** Data of participants baseline characteristics of both intervention group and control group

Authors	Mogahed (2020) [39]		Kilmartin (2019) [38]		Baxter (2018) [37]		Storz (2017) [36]		Bramlett (2014) [35]		Ridner (2013) [40]		Lau and Cheing (2010) [34]		Omar (2011) [21]	
Characteristics	I	C	I	C	I	C	I	C	I	C	I	C	I	C	I	C
Age (y)	48.4	48.33	63.64	59.6	57.9	64.3	56.12 ± 8.85	53.17 ± 11.42	54.3	66.1	66.4	67.5	50.9 ± 8.6	51.3 ± 8.9	54.76 ± 3.33	53.36 ± 3.56
BMI (kg/cm2), Mean	NR	NR	27.56	26.55	27.9	30.5	NR	NR	NR	NR	NR	NR	NR	NR	29.1 ± 6.6	25.6 ± 3.3
Dominant arm	NR	NR	NR	NR	5	3	NR	NR	NR	NR	5	10	NR	NR	20	19
Non-dominant arm	NR	NR	NR	NR	NR	NR	NR	NR	NR	NR	10	5	NR	NR	5	6
Received Radiotherapy	NR	NR	100% (11/11)	90% (9/10)	9	7	14 (82.4%)	16 (84.2%)	NR	NR	2 (14.3%)	2(13.3%)	90.90%	100%	10	9
Received Chemotherapy	NR	NR	90.9% (10/11)	90% (9/10)	8	6	13 (76.5%)	14 (73.7%)	NR	NR	1(7.1%)	1(6.7%)	54.60%	60%	10	9

*I* intervention Group

*C* control group

*NR* Not reported

### Characteristics of clinical trials

The essential features of the studies considered in this review are shown in Table 2. All publications were written in English and described how LLLT (PBM) helped people with breast cancer-related lymphedema. RCTs were published between 2010 and 2022 [21, 34–40] and the sample size for the selected eight research ranged from 14 to 50 female participants [21, 34–40]. All clinical studies looked at women diagnosed with post-mastectomy lymphedema between the age of 40 to 77. All the studies examined arm circumference/volume, with two of them also measuring pain severity [37, 39] and shoulder mobility [21, 36]. Traditional treatments such as manual lymphatic drainage [39, 40] and compression garments were compared to laser therapy [21, 37, 40]. In addition, Sackett's level of evidence for each clinical trial was examined for RCTs, and eight of the RCTs were rated as level 2, as shown in Table 2. [21, 34–40]. The RCTs had different follow-up periods. The participants were monitored for three months in three trials [36, 37, 39]. One research looked at the patients for a month [34], while another trial looked at them for four months [21]. Three trials concluded the therapy with a six-month follow-up period [35, 38, 40].

**Table 2:** Characteristics for eight included clinical trials

Authors [Years]	Subjects (n)	Level of Evidence	Study Design and Blinding type	Inclusion Criteria	Interventions	Follow-up sessions	Evaluation Outcomes	Final Findings
Mogahed et al. [2020] [39]	30 women, unilateral PML	Level 2	Single blinded, controlled trial	Stage 2 or 3 BCRL	1: Active laser (*n* = 15) 2: Placebo laser (*n* = 15) 3: MLD 4: Shoulder ROM 5: Pneumatic compression	Pre-Tx-3 mn	1: Arm volume 2: Shoulder pain	Laser group was found beneficial in reducing upper arm volume and decreasing shoulder pain as a post-mastectomy complication
Kilmartin et al. [2019] [38]	22 women, unilateral PML	Level 2	Double-blinded Randomized, placebo-controlled trial	Age ≥ 21 yr, Stage 2 or 3 unilateral lymphedema	1: Active laser + CDT (*n* = 11) 2: Placebo laser + CDT (n = 11)	Pre-Tx, 3,6,12 mn	1: Arm volume 2: Shoulder mobility	Laser group remarably decreases post-mastectomy arm volume and improve symptoms compared to control placebo group
Baxter et al. [2018] [37]	16 women, unilateral PML	Level 2	Double- blinded Randomized controlled trial	Age ≥ 18 yr, Diagnosis of BCRL	1: Active laser (*n* = 8) 2: Placebo laser (*n* = 8) 3: Pressure garment 4: Massage therapy & Exe	Pre-Tx, 6,12 wk	1: Arm circumference	Laser group seemed to have improve arm circumference, in general LLLT was considered to treat BCRL
Storz et al. [2017] [36]	40 women, unilateral PML	Level 2	Double- blinded Randomized, Placebo-controlled trial	≥ 3 months hx of BCRL	1: Active laser (*n* = 20) 2: Placebo laser (*n* = 20) 3: Daily limb exe 4: Skin care	Pre-Tx, 4,8,12 wk	1: Arm volume 2: Shoulder pain	Laser therapy may be effective in reduction of arm volume, but no solid results were seen
Bramlett et al. [2014] [35]	14 women, unilateral PML	Level 2	Double-blinded, Randomized placebo-controlled trial	Age ≥ 21 yr, unilateral BCRL	1: Active laser (*n* = 7) 2: Placebo laser (*n* = 7) CDT	Pre-Tx 3,6,12,18 mn	1: Arm circumference and volume	18-months of period suggested a decreasing trend in active laser group; Long-term follow-up appears to show that LLLT is useful
Ridner et al. [2013] [40]	46 women, unilateral PML	Level 2	Single blinded, Randomized controlled trial	Age ≥ 21 yr, Stage 1 or 2 lymphedema	1: Active laser (*n* = 15) 2: MLD group (*n* = 16) 3: Laser + MLD (*n* = 15) 4: Compression Bandage	Pre-Tx, Daily and weekly, post Tx	1: Arm circumference and volume	Active laser with bandaging was found to be effective, similarly laser with MLD also helped managed PML
Lau and cheing [2010] [34]	21 women, unilateral PML	Level 2	Single-blinded, Randomized controlled trial	Age ≥ 18 yr, undergone radical mastectomy	1: Active laser (*n* = 11) 2: Placebo laser (*n* = 10) 3:Lymphedema education	Pre-Tx,4wk post-Tx	1: Arm volume	Active laser group showed notable decrease in arm volume at the 4wk follow-up
Omar et al. [2011] [21]	50 women, unilateral PML	Level 2	Double-blinded, Randomized, Placebo-controlled trial	Stage 2 or 3 breast cancer	1: Active laser (*n* = 25) 2: Placebo laser (*n* = 25) 3: Limb exe 4: Skin protection 5: Pressure garment	Pre-Tx 4, 8, 12, 16 wk	1: Arm circumference 2: Shoulder mobility 3: Hand grip strength	Laser was found to be effective to manage arm volume, shoulder ROM and hand grip strength in women with PML

Representation in Table 2.

*Hx* history, *MLD* manual lymphatic drainage, *CDT* Complete decongestive therapy, *Pre-Tx* Pre Treatment, *Post-Tx* Post Treatment, *Wk* week, *Mn* month

### Methodological quality

Table 3 shows the quality evaluation scores of the eight studies considered. The RCTs' quality was judged using the PEDro criteria. Three of the eight RCT publications were already evaluated for methodological quality using the PEDro scale [21, 34, 40] So, two reviewers [35–39] independently analysed the other five papers using the PEDro scale. All the publications had a score greater than the cut-off of 5, suggesting that the research was of excellent quality. As a result, research scoring 5 to 9 on the Sacketts level of evidence was designated as level 2.

**Table 3:** The PEDro scale was used to assess the quality of the included RCTs

Authors	Eligibility criteria	Random allocation	Concealed allocation	Baseline comparability	Subject were Blinded	Therapist were Blinded	Assessor were Blinded	Less than 15% dropout	Intention-to -treat analysis	Statistical comparisons between groups	Data on point measures and variability	Final points (criteria one not summed)
Mogahed et al. [2020] [39]	Yes	Yes	No	Yes	Yes	No	No	Yes	No	Yes	Yes	**6**
Kilmartin et al. [2019] [38]	Yes	Yes	No	Yes	Yes	No	Yes	Yes	No	Yes	Yes	**7**
Baxter et al. [2018] [37]	Yes	Yes	Yes	Yes	Yes	No	Yes	Yes	Yes	Yes	Yes	**9**
Storz et al. [2017] [36]	Yes	Yes	No	Yes	Yes	No	Yes	Yes	No	Yes	Yes	**7**
Bramlett et al. [2014] [35]	Yes	Yes	No	Yes	Yes	No	Yes	Yes	No	Yes	Yes	**7**
Ridner et al. [2013] [40]	Yes	Yes	No	Yes	No	No	No	Yes	Yes	Yes	Yes	**6**
Lau and cheing [2010] [34]	Yes	Yes	No	Yes	Yes	No	No	Yes	No	Yes	Yes	**6**
Omar et al. [2011] [21]	Yes	Yes	No	Yes	Yes	No	Yes	Yes	No	Yes	Yes	**7**
Sub-Item score	8	8	1	8	7	0	5	8	2	8	8	

### Assessment of risk of bias for clinical trials

To improve quality of our systematic review, Cochrane risk of bias version 2 [30] tool was used. The results for Rob 2.0 figure are presented in supplementary file. We deemed eight trials [21, 34–40], out of two studies [21, 39] to be at low risk bias, two studies [34, 38] at high risk and four trials [35–37, 40] followed by unclear risk of bias. We came to conclusion that domain 1 (randomization process) for revised version of Rob was apparently fulfilled by every trials. Three studies [35, 38, 40] did not clearly mentioned details for domain 3 (Missing outcome data) therefore judged as having high-risk of bias. All studies [21, 34–40] represented with unclear risk of bias regarding domain 5 ( Selection of reported results).

### Efficacy of low-level laser therapy

As there was little published research in this field, just a few RCTs were included in this systematic review. Furthermore, due to a scarcity of data, only post-treatment and three long-term follow-up studies and other short-term follow-up studies were included in this systematic review. Table 4 summarizes the findings of the various studies.

**Table 4:** Results of RCTs included in subgroup analysis summarized

Authors	Arm circumference/volume		Pain severity		Shoulder mobility	
	**At the end of entire treatment**	**Follow-up (< 6 months)**	**At the end of entire treatment**	**Follow-up (< 6 months)**	**At the end of entire treatment**	**Follow-up (< 6 months)**
Mogahed et al. [2020] [39]	Strong	NR	Strong	NR	NR	NR
Kilmartin et al. [2019] [38]	Strong	Strong	Strong	NR	Strong	NR
Baxter et al. [2018] [37]	Strong	Weak	Strong	Strong	NR	NR
Storz et al. [2017] [36]	Weak	Weak	Strong	NR	NR	NR
Bramlett et al. [2014] [35]	Strong	Weak	NR	NR	NR	NR
Ridner et al. [2013] [40]	Weak	NR	NR	NR	NR	NR
Lau and Cheing [2010] [34]	Strong	Weak	NR	NR	NR	NR
Omar et al. [2011] [21]	Strong	Strong	NR	NR	Strong	Weak

### Laser therapy for arm circumference

Four trials have demonstrated the importance of low-level laser treatment in reducing the disparity in arm circumference between affected and unaffected limbs [21, 35, 37, 40]. The most popular method for measuring arm circumference was to wrap a non-stretch tape around the extremity, leaving no slack or indentation in the tissue. Measuring points were made from the ulnar styloid process to the axilla [21, 37, 40]. According to a trial, long-term investigations revealed a statistically significant reduction in arm circumference [35]. Two RCTs found that using LLLT versus placebo lasers offered moderate evidence [21, 38]. At 6- and 12-weeks after randomization, one high-quality research found that patients were satisfied with the use of LLLT for BCRL [37].

### Laser therapy for arm volume

The reduction of indifference in arm volume between the affected and sound limb was determined in four high-quality clinical trials [35, 36, 38, 39] that evaluated outcomes of LLLT in patients with post-mastectomy lymphedema. Although the standardized method varied among the RCTs, one study [34] used a tank volumeter to assess the arm volume, which showed a massive reduction in volume by around 28% in the laser group, whereas in contrast; the control showed 6% increase in arm volume by the end of 4-week follow-up. Two trials [36, 40] with moderate evidence found a statistically significant effect of laser therapy regarding decreasing arm volume. However, no difference was found in such comparisons between the two groups at the end of treatment. Finally, one high-quality study [39] provided strong evidence of the comparison between affected and sound limbs when measured at the start and end of treatment.

### Laser therapy for shoulder mobility

Two clinical studies [21, 38] found that using LLLT for breast cancer-related lymphedema improved patients' ranges of motion at the shoulder joint. A plastic goniometer was utilized in one high-quality study [21] to evaluate active ROM for (shoulder flexion, shoulder abduction, and external rotation), resulting in a substantial increase in ROM for two movements (shoulder flexion and abduction) at the affected arm after 8–12 weeks. However, these studies reported that there was no increase in external rotation. One low-quality RCT [38] found no convincing evidence that the use of low-level laser treatment for BCRL improved shoulder mobility.

### Laser therapy for pain severity

The change in the difference in pain severity was demonstrated in two RCTs [36, 39]. One trial measured pain severity on a 10-point scale [36], and the other trial used a 0–100 mm visual analogue scale [39], which was converted into a 10-point scale. One high-quality study [36] provided strong evidence that LLLT was influential in reducing pain by 50% at the end of treatment compared to the beginning of the session. Similarly, another trial [39] provided moderate evidence supporting laser therapy to reduce pain for patients with BCRL.

### Application of laser therapy for BCRL

Data extracted regarding the parameters of laser therapy from eight studies are displayed in Table 5. Major studies did not reasonably follow WALT guidelines [33] for the use of laser therapy. The wavelength commonly used was 904-nm, given in 4/8 studies [21, 35, 38, 40], whereas one trial used a combination of two wavelengths [34], and two studies used 980-nm for the treatment [36, 37]. The standard application zone was the chest wall and axillary area, or over the affected arm. Each experiment included an estimated 1 min of laser energy per location. The treatment session regimen was generally three times each week, with length ranging from four to twelve weeks.

**Table 5:** Laser protocols used in clinical trials

RCTs	Laser Unit	Wavelength (nm)	Treatment Sessions	Application Zone
Mogahed et al. [39]	Bravo Terza Serie, Model D	905	36 sessions, 3times/week/12 weeks	Scanner 50 cm perpendicular to treatment area
Kilmartin et al. [38]	Rian-Corp Laser	904	8–16 sessions,	1 min/spot/10 sites in Axilla and a portion of chest wall
Baxter et al. [37]	Light-Force EX, LTS-1500	980	12 sessions, 2times/week/6 weeks	10 points from axilla to wrist on affected side
Storz et al. [36]	TIMELAS Vital	980	8 sessions, 2times/week/4 weeks	Spot covered 4.9cm2, 10 min each session
Bramlett et al. [35]	Rian-Corp Laser	904	16 sessions, 2time/week/8 weeks	1 min/10spots over the axillary region
Ridner et al. [40]	Rian-Corp Laser	904	10 Sessions, 20 min each	20-30 s/spot, over the area to be treated
Lau and Cheing [34]	Comby 3 Terza Serie, Model D	808	12 sessions, 3times/week/4 weeks	Entire Axillary Region (144cm2)
		905 × 2		
Omar et al. [21]	Rian-Corp, Ga-As Laser	904	36 Sessions,3times/week/12 weeks	2 min/spot, 20 min, over the affected arm

## Dicussion

In the last two decades, LLLT has attracted much attention. It has been used to treat lymphedema and a variety of other ailments, including musculoskeletal issues. In addition, since the LLLT has been employed in clinical settings, it has improved patient satisfaction following treatment. When compared to other procedures used to treat postmastectomy lymphedema, laser therapy has, according to the results of RCTs, not only shown to be successful but also time efficient. This review sought to determine the best outcomes for the therapeutic application of low-level laser therapy since it supported the conclusion of previously published literature [20], and demonstrated the efficiency of LLLT in the management of BCRL.

Even though, manual lymphatic drainage (MLD) [41], when compared to laser therapy, has proved a beneficial therapeutic and rehabilitative strategy. Rehabilitative exercise, complex decongestive therapy (CDT), and multi-layer bandaging for compression also helps to reduce arm volume [42]. however, when these interventions are combined with low-level laser treatment, a significant difference in arm volume is obtained.

As a result, the primary goal of this systematic review was to see how effective LLLT (PBM) is for BCRL. Once all studies were qualified to be included in the inclusion criteria, eight published RCTs were chosen based on the best evidence synthesis. This systematic review assesses recent RCTs on the efficacy of LLLT that were published after 2017 and had not previously been included in any systematic review. As a result, we tried to incorporate freshly published research and past trials in our analysis.

Secondly, we focused on conducting a systematic review on long-term follow-up [35, 38, 40], with a duration of more than six months, to survey whether that would make a difference in the results compared with the short-term follow-up to treat females with BCRL. Thirdly, this review examined the quality of RCTs by assessing them on Sackett’s level of evidence [32]. Eight of these randomized controlled trials were graded on level 2. The treatment approaches used, and their respective results in this randomized controlled trial were apparent to some extent. However, none of the studies stated well-defined results to be marked at level 1 on Sackett’s level of evidence.

With the sheer random assignment of individuals and the control of external factors, the reported trials offered the greatest evidence for treatment effectiveness. This has strengthened the experimental design and made the results less biased. (Four high quality trials) supporting LLLT (PBM) over placebo laser group showed a reduction in arm circumference at four weeks follow up. In comparison, this review provided moderate evidence in support of LLLT for the decline in arm volume seen in (four high quality trials), which eventually favoured the laser therapy group over the placebo group and (two low quality trials) supported laser therapy over the placebo group when compared for affected arm shoulder mobility.

Considering RCTs are the high benchmark in the medical field, the findings of the eight RCTs on the usefulness of Laser therapy were assessed in a critical appraisal (PBM). The methodological quality of the RCTs under investigation was graded as 'strong' on the PEDro scale, with four trials receiving a score of 7/10 and one study receiving a score of 9/10. Despite this, clinical variation in treatment techniques was evident throughout all eight studies. Unfortunately, a solid comparison to establish the treatment regime was unattainable due to the small number of published publications.

Despite WALT [33] unambiguous wordings suggesting a regulated variable for the wavelength, appropriate dose, and duration of laser therapy, the authors could not describe the treatment parameters completely in RCTs. This is not unusual, as other reviewers have also pointed out these flaws [43, 44]. Furthermore, variations in treatment processes, methodologies, application sites, and variability among the factors make it difficult to pool information on the usage of LLLT (PBM). Unless a comparable WALT suggestion is adequately followed, this will contradict outcomes.

All our reviewed RCTs, commonly wavelengths between (808 nm and 980 nm), and energy densities between 1.5–4.89 per centimetre squared (J/cm2) were reported in included studies. Similarly, depending on the location of the tendon, efficient power concentrations for tendinopathy vary between 1.8 to 19.2 J/cm2 [43]. Treatment sessions were typically four weeks long. However, trials with extended durations are also discussed in this review to assess the efficacy of Laser therapy for BCRL.

To reduce the element of bias, our systematic review closely adhered to a robust strategy. To begin, in terms of external validity, the review process followed the Preferred Reporting Items for Systematic Reviews and Meta-Analysis 2020 (PRISMA) recommendations [23]. Second, Sackett's level of evidence was utilised to describe the research based on the methodological quality and validity of their design [32]. Third, subgroup analyses were conducted to assess clinical heterogeneity for result synthesis. Finally, the review's results were derived from eight high-quality techniques research.

### Limitations

This systematic review's major limitation is that it primarily comprises of free online papers, not paid or grey literature. Moreover, some of the RCTs that were examined had shorter follow-up durations due to the absence of published literature on the long-term effects of low-level laser therapy; therefore, a review with a all RCTs with longer follow-up may anticipate positive outcomes. Time constraints hindered the conduct of a meta-analysis, which would have improved understanding and helped this field form new trends. This evaluation concludes that further well-designed studies are required to fully confirm the efficacy of laser therapy in the treatment of lymphedema in breast cancer patients.

## Conclusions

This systematic review revealed that LLLT (PBM) in a reduction in arm circumference and volume was more successful than improving shoulder mobility and pain. Data covey that the application of laser therapy (PBM) may be a positive approach for females with PML. Due to the scarcity of data, there is a strong need for well-conducted studies in this field.

## Supplementary Information

**Additional file 1.** 

## Data Availability

All information gathered or analysed during this study are given in this article [and its additional files].

## Abbreviations

BCRL: Breast cancer related lymphedema
LLLT: Low-level laser therapy
PML: Post-mastectomy lymphedema
PBM: Photo-biomodulation
PRISMA: Preferred Reporting Items for Systematic Reviews and Meta-analysis
RCT: Randomized Controlled Trials
WALT: World Association for laser therapy
ATP: Adenosine triphosphate
REDOX: Reduction–oxidation reaction
