# Supplementary material for: Clinical application of low-level laser therapy (Photo-biomodulation therapy) in the management of breast cancer-related lymphedema: a systematic review
Source: BMC Cancer. 2022 Aug 30;22:937. doi: 10.1186/s12885-022-10021-8 (PMC9426030; doi:10.1186/s12885-022-10021-8)
Supplement: Supplementary file 1 — Additional file 1. [file 12885_2022_10021_MOESM1_ESM.docx]

**1.1 Literature Search**

**Literature Search for each database-**Search strategy

1. PubMed (from inception to October 15^th^, 2021)

(1) laser [MeSH Terms] 188,718

(2) laser therapy [MeSH Terms] 45,808

(3) laser light [MeSH Terms] 35,103

(4) laser light therapy [MeSH Terms] 13,193

(5) low level laser [MeSH Terms] 7,291

(6) cold laser [MeSH Terms] 1,915

(7) cold laser therapy [MeSH Terms] 5,771

(8) low energy laser MeSH Terms] 5,178

(9) low level laser therapy [MeSH Terms] 6,592

(10) low energy laser therapy [MeSH Terms] 1,341

(11) low intensity laser [MeSH Terms] 3,557

(12) photo-biomodulation [MeSH Terms] 1,961

(13) lymphedema [MeSH Terms] 8,256

(14) breast cancer lymphedema [MeSH Terms] 2,041

(15) lymphoedema [MeSH Terms] 8,256

(16) swelling [MeSH Terms] 117,781

(17) edema [MeSH Terms] 75,862

(18) oedema MeSH Terms] 75,862

(19) breast cancer [MeSH Terms] 244,147

(20) or/(1)-(12) 189,189

(21) (9) and (19) and (13) 24

1. PEDro Physiotherapy Evidence Database (from inception to October 15^th^, 2021)

(1) Title: Laser 299

(2) Title: Laser therapy 206

(3) Title: Low level laser 147

(4) Title: Low level laser therapy 126

(5) Title: Cold laser 1

(6) Title: Low energy laser 4

(7) Title: Low intensity laser 14

(8) Title: Photo-biomodulation 58

(9) Title: Edema 36

(10) Title: oedema 10

(11) Title: swelling 11

(12) Title: lymphedema 150

(13) Title: lymphoedema 32

(14) Title: Breast cancer 820

(15) (3) and (14) and (12) 3

1. Cochrane Library (from inception to October 15^th^, 2021)

(1) Laser 21287

(2) Laser therapy 11463

(3) Low energy laser 786

(4) Low intensity laser 869

(5) Cold laser 359

(6) Cold laser therapy 191

(7) Low level laser 3882

(8) Low level laser therapy 3548

(9) photo-biomodulation 757

(10) edema 20190

(11) oedema 20190

(12) lymphedema 1545

(13) lymphoedema 1545

(14) swelling 9053

(15) breast cancer 39919

(16) (8) and (15) and (12) 18

1. Medline National Library of Medicine (NLM) (from inception to October 15^th^, 2021)

**Same results were driven as PubMed**

**1.2 Studies excluded after full-text assessment**

**Excluded articles after duplicates removal (n=53)**

| **References** | **Reason for Exclusion** |
| --- | --- |
| Kaviani A, Fateh M, Yousefi Nooraie R, Alinagi-zadeh MR, Ataie-Fashtami L**. Low-level laser therapy in management of postmastectomy lymphedema.** Lasers Med Sci. 2006 Jul;21(2):90-4. | Not a clinical trial |
| Jang DH, Song DH, Chang EJ, Jeon JY. **Anti-inflammatory and lymphangiogenetic effects of low-level laser therapy on lymphedema in an experimental mouse tail model.** Lasers Med Sci. 2016 Feb;31(2):289-96. | Not a clinical trial |
| Dirican A, Andacoglu O, Johnson R, McGuire K, Mager L, Soran A. **The short-term effects of low-level laser therapy in the** **management of breast-cancer-related lymphedema.** Support Care Cancer. 2011 May;19(5):685-90. | Not a clinical trial |
| Carati CJ, Anderson SN, Gannon BJ, Piller NB. **Treatment of postmastectomy lymphedema with low-level laser therapy: a double blind, placebo-controlled trial**. Cancer. 2003 Sep 15;98(6):1114-22. | Published before 2010 |
| Kozanoglu E, Basaran S, Paydas S, Sarpel T. **Efficacy of pneumatic compression and low-level laser therapy in the treatment of** **postmastectomy lymphoedema**: a randomized controlled trial. Clin Rehabil. 2009 Feb;23(2):117-24. | Irrelevant intervention |
| Mayrovitz HN, Davey S. **Changes in tissue water and indentation resistance of lymphedematous limbs accompanying low level** **laser therapy (LLLT) of fibrotic skin.** Lymphology. 2011 Dec;44(4):168-77. | Irrelevant population |
| Balci FL, DeGore L, Soran A: **Breast cancer-related lymphedema in elderly patients**. *Topics in Geriatric Rehabilitation* 2012, 28(4):243-253. | Review |
| Brorson H: **Liposuction gives complete reduction of chronic large arm lymphedema after breast cancer**. *Acta Oncol* 2000, 39(3):407-420. | Irrelevant intervention |
| Cebicci MA, Sutbeyaz ST, Goksu SS, Hocaoglu S, Oguz A, Atilabey A: **Extracorporeal shock wave therapy for breast cancer-related lymphedema: A pilot study**. *Arch Phys Med Rehabil* 2016, 97(9):1520-1525. | Irrelevant intervention |
| Chau N, Harris S: **Practices and opinions of physiotherapists treating patients with breast cancer-related lymphedema**. *Physiother Can* 2002, 54(3):156-163. | Not a clinical trial |
| Costa MM, Silva SB, Quinto ALP, Pasquinelli PFS, dos Santos VD, Santos GD, Veiga DF: **Phototherapy 660 nm for the prevention of radiodermatitis in breast cancer patients receiving radiation therapy: Study protocol for a randomized controlled trial**. *Trials* 2014, 15. | Study protocol |
| Cheng MH, Chen SC, Henry SL, Tan BK, Lin MCY, Huang J: **Vascularized groin lymph node flap transfer for postmastectomy upper limb lymphedema: Flap anatomy, recipient sites, and outcomes**. *Plast Reconstr Surg* 2013, 131(6):1286-1298. | Irrelevant intervention |
| Fu MR, Ridner SH, Armer J: **Post-breast cancer lymphedema Part 2**. *Am J Nurs* 2009, 109(8):34-41. | Review |
| Afifi L, Maranda EL, Zarei M, Delcanto GM, Falto-Aizpurua L, Kluijfhout WP, Jimenez JJ. **Low-level laser therapy as a** **treatment for androgenetic alopecia.** Lasers Surg Med. 2017 Jan;49(1):27-39. | Irrelevant population |
| Mussttaf RA, Jenkins DFL, Jha AN. **Assessing the impact of low level laser therapy (LLLT) on biological systems: a** **review**. Int J Radiat Biol. 2019 Feb;95(2):120-143. | Irrelevant population |
| Avci P, Gupta A, Sadasivam M, Vecchio D, Pam Z, Pam N, Hamblin MR**. Low-level laser (light) therapy (LLLT) in skin: stimulating, healing, restoring.** Semin Cutan Med Surg. 2013 Mar;32(1):41-52. | Review |
| Smoot B, Chiavola-Larson L, Lee J, Manibusan H, Allen DD. **Effect of low-level laser therapy on pain and swelling in women** **with breast cancer-related lymphedema:** a systematic review and meta-analysis. J Cancer Surviv. 2015 Jun;9(2):287-304. | Review |
| Powell K, Low P, McDonnell PA, Laakso EL, Ralph SJ: **The effect of laser irradiation on proliferation of human breast carcinoma, melanoma, and immortalized mammary epithelial cells**. *Photomed Laser Surg* 2010, 28(1):115-123. | Not a clinical trial |
| Wang Y, Ge Y, Xing W, Liu J, Wu J, Lin H, Lu Y. **The effectiveness and safety of low-level laser therapy on breast cancer-related** **lymphedema: An overview and update of systematic reviews.** Lasers Med Sci. 2022 Apr;37(3):1389-1413. | Review |
| Poage E, Singer M, Armer J, Poundall M, Shellabarger J: **Demystifying lymphedema: Development of the lymphedema putting evidence into practice (R) card**. *Clin J Oncol Nurs* 2008, 12(6):951-964. | Review |
| Eisner A, O'Malley JP, Incognito LJ, Toomey MD, Samples JR: **Small optic cup sizes among women using tamoxifen: Assessment with scanning laser ophthalmoscopy**. *Curr Eye Res* 2006, 31(4):367-379. | Irrelevant intervention |
| Lin J, Jandial R, Nesbit A, Badie B, Chen MK: **Current and emerging treatments for brain metastases**. *Oncology-NY* 2015, 29(4):250-257. | Irrelevant population |
| Okuno T, Kato S, Hatakeyama Y, Okajima J, Maruyama S, Sakamoto M, Mori S, Kodama T: **Photothermal therapy of tumors in lymph nodes using gold nanorods and near-infrared laser light**. *J Control Release* 2013, 172(3):879-884. | Irrelevant population |
| Mihara M, Hara H, Hayashi Y, Iida T, Araki J, Yamamoto T, Todokoro T, Narushima M, Murai N, Koshima I: **Upper-limb lymphedema treated aesthetically with lymphaticovenous anastomosis using indocyanine green lymphography and noncontact vein visualization**. *J Reconstr Microsurg* 2012, 28(5):327-332. | Irrelevant intervention |
| Moseley AL, Carati CJ, Piller NB: **A systematic review of common conservative therapies for arm lymphoedema secondary to breast cancer treatment**. *Ann Oncol* 2007, 18(4):639-646. | Review |
| e Lima MT, e Lima JG, de Andrade MF, Bergmann A: **Low-level laser therapy in secondary lymphedema after breast cancer: Systematic review**. *Lasers Med Sci* 2014, 29(3):1289-1295. | Review |
| Perbeck L, Celebioglu F, Svensson L, Danielsson R: **Lymph circulation in the breast after radiotherapy and breast conservation**. *Lymphology* 2006, 39(1):33-40. | Irrelevant population |
| Lai CC, Chen SY, Tu YK, Ding YW, Lin JJ. **Effectiveness of low level laser therapy versus cryotherapy in cancer patients** **with oral mucositis**: Systematic review and network meta-analysis. Crit Rev Oncol Hematol. 2021 Apr;160:103276. | Irrelevant population |
| Finfter O, Avni B, Grisariu S, Haviv Y, Nadler C, Rimon O, Zadik Y. **Photobiomodulation (low-level laser) therapy for** **immediate pain relief of persistent oral ulcers in chronic graft-versus-host disease**. Support Care Cancer. 2021 Aug;29(8):4529-4534. | Irrelevant population |
| Hasenoehrl T, Palma S, Ramazanova D, Kölbl H, Dorner TE, Keilani M, Crevenna R. **Resistance exercise and breast** **cancer-related lymphedema-a systematic review update and meta-analysis**. Support Care Cancer. 2020 Aug;28(8):3593-3603. | Review |
| Pajero Otero V, García Delgado E, Martín Cortijo C, Romay Barrero HM, de Carlos Iriarte E, Avendaño-Coy J. **Kinesio taping versus compression garments for treating breast cancer-related lymphedema: a randomized, cross-over,** **controlled trial.** Clin Rehabil. 2019 Dec;33(12):1887-1897. | Irrelevant intervention |
| Pasyar N, Barshan Tashnizi N, Mansouri P, Tahmasebi S. **Effect of yoga exercise on the quality of life and upper extremity volume among women with breast cancer related lymphedema: A pilot study**. Eur J Oncol Nurs. 2019 Oct;42:103-109. | Irrelevant intervention |
| Sanal-Toprak C, Ozsoy-Unubol T, Bahar-Ozdemir Y, Akyuz G. **The efficacy of intermittent pneumatic compression as a substitute for manual lymphatic drainage in complete decongestive therapy in the treatment of breast cancer related** **lymphedema.** Lymphology. 2019;52(2):82-91. | Irrelevant intervention |
| Sanders JM, Butt L, Clark A, Williams J, Padgen M, Leung E, Keely P, Condeelis JS, Aguirre-Ghiso J, Castracane J: **A BioMEMS device for the study of mechanical properties of cells**. In: *Microfluidics, Biomems, and Medical Microsystems Xiii. Volume 9320*, edn. Edited by Gray BL, Becker H; 2015. | Not a clinical trial |
| Bloomquist K, Karlsmark T, Christensen KB, Adamsen L. **Heavy resistance training and lymphedema: prevalence of** **breast cancer-related lymphedema in participants of an exercise intervention utilizing heavy load resistance training**. Acta Oncol. 2014 Feb;53(2):216-25. | Not a clinical trial |
| Rodrick JR, Poage E, Wanchai A, Stewart BR, Cormier JN, Armer JM: **Complementary, alternative, and other noncomplete decongestive therapy treatment methods in the management of lymphedema: A systematic search and review**. *Pm&R* 2014, **6**(3):250-274. | Review |
| Krok-Schoen JL, Oliveri JM, Kurta ML, Paskett ED: **Breast cancer-related lymphedema: Risk factors, prevention, diagnosis and treatment**. *Breast Cancer Manag* 2015, **4**(1):41-51. | Review |
| Cheema BS, Kilbreath SL, Fahey PP, Delaney GP, Atlantis E. **Safety and efficacy of progressive resistance training in** **breast cancer: a systematic review and meta-analysis**. Breast Cancer Res Treat. 2014 Nov;148(2):249-68. | Review |
| Mok J, Brown MJ, Akam EC, Morris MA. **The lasting effects of resistance and endurance exercise interventions on** **breast cancer patient mental wellbeing and physical fitness**. Sci Rep. 2022 Mar 3;12(1):3504. | Irrelevant intervention |
| Shousha T, Alayat M, Moustafa I. **Effects of low-level laser therapy versus soft occlusive splints on mouth opening and surface electromyography in females with temporomandibular dysfunction: A randomized-controlled study.** PLoS One. 2021 Oct 1;16(10):e0258063. | Irrelevant population |
| Herpich CM, Leal-Junior EC, Amaral AP, Tosato Jde P, Glória IP, Garcia MB, Barbosa BR, El Hage Y, Arruda ÉE, Gomes CÁ, Rodrigues MS, de Sousa DF, de Carvalho Pde T, Bussadori SK, Gonzalez Tde O, Politti F, Biasotto-Gonzalez DA. **Effects of phototherapy on muscle activity and pain in individuals with temporomandibular disorder: a study protocol for a randomized controlled trial.** Trials. 2014 Dec 16;15:491. | Study protocol |
| Sanders JM, Butt L, Clark A, Williams J, Padgen M, Leung E, Keely P, Condeelis JS, Aguirre-Ghiso J, Castracane J: **A BioMEMS device for the study of mechanical properties of cells**. In: *Microfluidics, Biomems, and Medical Microsystems Xiii. Volume 9320*, edn. Edited by Gray BL, Becker H; 2015. | Not a clinical trial |
| Stanton AWB, Levick JR, Mortimer PS: **Cutaneous vascular control in the arms of women with postmastectomy oedema**. *Exp Physiol* 1996, **81**(3):447-464. | Irrelevant intervention |
| Maia ML, Bonjardim LR, Quintans Jde S, Ribeiro MA, Maia LG, Conti PC. **Effect of low-level laser therapy on pain levels** **in patients with temporomandibular disorders: a systematic review**. J Appl Oral Sci. 2012 Nov-Dec;20(6):594-602. | Review |
| Cetiner S, Kahraman SA, Yücetaş S. **Evaluation of low-level laser therapy in the treatment of temporomandibular** **disorders.** Photomed Laser Surg. 2006 Oct;24(5):637-41. | Irrelevant population |
| Bao T, Iris Zhi W, Vertosick EA, Li QS, DeRito J, Vickers A, Cassileth BR, Mao JJ, Van Zee KJ. **Acupuncture for breast** **cancer-related lymphedema: a randomized controlled trial.** Breast Cancer Res Treat. 2018 Jul;170(1):77-87. | Irrelevant intervention |
| Ezzo J, Manheimer E, McNeely ML, Howell DM, Weiss R, Johansson KI, Bao T, Bily L, Tuppo CM, Williams AF, Karadibak D. **Manual lymphatic drainage for lymphedema following breast cancer treatment.** Cochrane Database Syst Rev. 2015 May 21;(5):CD003475. | Irrelevant intervention |
| De Vrieze T, Vos L, Gebruers N, Tjalma WAA, Thomis S, Neven P, Nevelsteen I, De Groef A, Vandermeeren L, Belgrado JP, Devoogdt N. **Protocol of a randomised controlled trial regarding the effectiveness of fluoroscopy-guided manual lymph drainage for the treatment of breast cancer-related lymphoedema** (EFforT-BCRL trial). Eur J Obstet Gynecol Reprod Biol. 2018 Feb;221:177-188. | Study protocol |
| Rogan S, Taeymans J, Luginbuehl H, Aebi M, Mahnig S, Gebruers N: **Therapy modalities to reduce lymphoedema in female breast cancer patients: A systematic review and meta-analysis**. *Breast Cancer Res Treat* 2016, **159**(1):1-14. | Review |
| Suarez Y, Gonzalez L, Cuadrado A, Berciano M, Lafarga M, Munoz A: **Kahalalide F, a new marine-derived compound, induces oncosis in human prostate and breast cancer cells**. *Mol Cancer Ther* 2003, **2**(9):863-872. | Irrelevant intervention |
| Brorson H, Svensson H: **Skin blood flow of the lymphedematous arm before and after liposuction**. *Lymphology* 1997, 30(4):165-172. | Irrelevant intervention |
| Kuz'mina EG, Degtiareva AA, Doroshenko LN, Rogova NM, Zorina LN: **[Immunologic indices of the blood and interstitial fluid in the evaluation of the treatment of secondary edemas of the upper extremities]**. *Med Radiol (Mosk)* 1990, **35**(5):18-21. | Irrelevant population |
| Devoogdt N, Geraerts I, Van Kampen M, De Vrieze T, Vos L, Neven P, Vergote I, Christiaens MR, Thomis S, De Groef A. **Manual lymph drainage may not have a preventive effect on the development of breast cancer-related lymphoedema** **in the long term: a randomised trial.** J Physiother. 2018 Oct;64(4):245-254. | Irrelevant intervention |

**1.3 DOI provided for included references (where available)**

1. Pinto AC, de Azambuja E. Improving quality of life after breast cancer: dealing with symptoms. Maturitas. 2011 Dec;70(4):343-8. doi: 10.1016/j.maturitas.2011.09.008. Epub 2011 Oct 19. PMID: 22014722.
2. Shibuya K, Mathers CD, Boschi-Pinto C, Lopez AD, Murray CJ. Global and regional estimates of cancer mortality and incidence by site: II. Results for the global burden of disease 2000. BMC Cancer. 2002 Dec 26;2:37. doi: 10.1186/1471-2407-2-37. Epub 2002 Dec 26. PMID: 12502432; PMCID: PMC149364.
3. Clark B, Sitzia J, Harlow W. Incidence and risk of arm oedema following treatment for breast cancer: a three-year follow-up study. QJM. 2005 May;98(5):343-8. doi: 10.1093/qjmed/hci053. Epub 2005 Apr 8. PMID: 15820971.
4. Olsson Möller U, Beck I, Rydén L, Malmström M. A comprehensive approach to rehabilitation interventions following breast cancer treatment - a systematic review of systematic reviews. BMC Cancer. 2019 May 20;19(1):472. doi: 10.1186/s12885-019-5648-7. PMID: 31109309; PMCID: PMC6528312.
5. Armer JM, Radina ME, Porock D, Culbertson SD. Predicting breast cancer-related lymphedema using self-reported symptoms. Nurs Res. 2003 Nov-Dec;52(6):370-9. doi: 10.1097/00006199-200311000-00004. PMID: 14639083.
6. Fu MR, Rosedale M. Breast cancer survivors' experiences of lymphedema-related symptoms. J Pain Symptom Manage. 2009 Dec;38(6):849-59. doi: 10.1016/j.jpainsymman.2009.04.030. PMID: 19819668; PMCID: PMC2795115.
7. De Vrieze T, Gebruers N, Tjalma WA, Nevelsteen I, Thomis S, De Groef A, Dams L, Van der Gucht E, Belgrado JP, Vandermeeren L, Devoogdt N. What is the best method to determine excessive arm volume in patients with breast cancer-related lymphoedema in clinical practice? Reliability, time efficiency and clinical feasibility of five different methods. Clin Rehabil. 2019 Jul;33(7):1221-1232. doi: 10.1177/0269215519835907. Epub 2019 Mar 18. PMID: 30880473.
8. de Sire A, Losco L, Cigna E, Lippi L, Gimigliano F, Gennari A, Cisari C, Chen HC, Fusco N, Invernizzi M. Three-dimensional laser scanning as a reliable and reproducible diagnostic tool in breast cancer related lymphedema rehabilitation: a proof-of-principle study. Eur Rev Med Pharmacol Sci. 2020 Apr;24(8):4476-4485. doi: 10.26355/eurrev_202004_21030. PMID: 32373985.
9. Invernizzi M, Runza L, De Sire A, Lippi L, Blundo C, Gambini D, Boldorini R, Ferrero S, Fusco N. Integrating Augmented Reality Tools in Breast Cancer Related Lymphedema Prognostication and Diagnosis. J Vis Exp. 2020 Feb 6;(156). doi: 10.3791/60093. PMID: 32090996.
10. Oremus M, Dayes I, Walker K, Raina P. Systematic review: conservative treatments for secondary lymphedema. BMC Cancer. 2012 Jan 4;12:6. doi: 10.1186/1471-2407-12-6. PMID: 22216837; PMCID: PMC3320521.
11. Moseley AL, Carati CJ, Piller NB. A systematic review of common conservative therapies for arm lymphoedema secondary to breast cancer treatment. Ann Oncol. 2007 Apr;18(4):639-46. doi: 10.1093/annonc/mdl182. Epub 2006 Oct 3. PMID: 17018707.
12. Nouri K, Jimenez GP, Harrison-Balestra C, Elgart GW. 585-nm pulsed dye laser in the treatment of surgical scars starting on the suture removal day. Dermatol Surg. 2003 Jan;29(1):65-73; discussion 73. doi: 10.1046/j.1524-4725.2003.29014.x. PMID: 12534515.
13. Lievens PC. The effect of a combined HeNe and i.r. laser treatment on the regeneration of the lymphatic system during the process of wound healing. Lasers in Medical Science. 1991;6(2):193-9. (DOI Not availible)
14. Jang DH, Song DH, Chang EJ, Jeon JY. Anti-inflammatory and lymphangiogenetic effects of low-level laser therapy on lymphedema in an experimental mouse tail model. Lasers Med Sci. 2016 Feb;31(2):289-96. doi: 10.1007/s10103-015-1854-y. Epub 2015 Dec 29. PMID: 26714983.
15. Assis L, Moretti AI, Abrahão TB, de Souza HP, Hamblin MR, Parizotto NA. Low-level laser therapy (808 nm) contributes to muscle regeneration and prevents fibrosis in rat tibialis anterior muscle after cryolesion. Lasers Med Sci. 2013 May;28(3):947-55. doi: 10.1007/s10103-012-1183-3. Epub 2012 Aug 17. PMID: 22898787; PMCID: PMC3521873.
16. Karu TIHL. Ten lectures on basic science of laser phototherapy. Gr??ngesberg: Prima Books; 2007. (DOI Not available)
17. Hou JF, Zhang H, Yuan X, Li J, Wei YJ, Hu SS. In vitro effects of low-level laser irradiation for bone marrow mesenchymal stem cells: proliferation, growth factors secretion and myogenic differentiation. Lasers Surg Med. 2008 Dec;40(10):726-33. doi: 10.1002/lsm.20709. PMID: 19065562.
18. Saygun I, Karacay S, Serdar M, Ural AU, Sencimen M, Kurtis B. Effects of laser irradiation on the release of basic fibroblast growth factor (bFGF), insulin like growth factor-1 (IGF-1), and receptor of IGF-1 (IGFBP3) from gingival fibroblasts. Lasers Med Sci. 2008 Apr;23(2):211-5. doi: 10.1007/s10103-007-0477-3. Epub 2007 Jul 10. PMID: 17619941.
19. Rocha Júnior AM, Vieira BJ, de Andrade LC, Aarestrup FM. Low-level laser therapy increases transforming growth factor-beta2 expression and induces apoptosis of epithelial cells during the tissue repair process. Photomed Laser Surg. 2009 Apr;27(2):303-7. doi: 10.1089/pho.2008.2277. PMID: 19382837.
20. Baxter GD, Liu L, Petrich S, Gisselman AS, Chapple C, Anders JJ, Tumilty S. Low level laser therapy (Photobiomodulation therapy) for breast cancer-related lymphedema: a systematic review. BMC Cancer. 2017 Dec 7;17(1):833. doi: 10.1186/s12885-017-3852-x. PMID: 29216916; PMCID: PMC5719569.
21. Ahmed Omar MT, Abd-El-Gayed Ebid A, El Morsy AM. Treatment of post-mastectomy lymphedema with laser therapy: double blind placebo control randomized study. J Surg Res. 2011 Jan;165(1):82-90. doi: 10.1016/j.jss.2010.03.050. Epub 2010 Apr 18. PMID: 20538293.
22. Carati CJ, Anderson SN, Gannon BJ, Piller NB. Treatment of postmastectomy lymphedema with low-level laser therapy: a double blind, placebo-controlled trial. Cancer. 2003 Sep 15;98(6):1114-22. doi: 10.1002/cncr.11641. Erratum in: Cancer. 2003 Dec 15;98(12):2742. PMID: 12973834.
23. Moher D, Liberati A, Tetzlaff J, Altman DG; PRISMA Group. Preferred reporting items for systematic reviews and meta-analyses: the PRISMA statement. BMJ. 2009 Jul 21;339:b2535. doi: 10.1136/bmj.b2535. PMID: 19622551; PMCID: PMC2714657.
24. Panic N, Leoncini E, de Belvis G, Ricciardi W, Boccia S. Evaluation of the endorsement of the preferred reporting items for systematic reviews and meta-analysis (PRISMA) statement on the quality of published systematic review and meta-analyses. PLoS One. 2013 Dec 26;8(12):e83138. doi: 10.1371/journal.pone.0083138. PMID: 24386151; PMCID: PMC3873291.
25. Schardt C, Adams MB, Owens T, Keitz S, Fontelo P. Utilization of the PICO framework to improve searching PubMed for clinical questions. BMC Med Inform Decis Mak. 2007 Jun 15;7:16. doi: 10.1186/1472-6947-7-16. PMID: 17573961; PMCID: PMC1904193.
26. Verhagen AP, de Vet HC, de Bie RA, Kessels AG, Boers M, Bouter LM, Knipschild PG. The Delphi list: a criteria list for quality assessment of randomized clinical trials for conducting systematic reviews developed by Delphi consensus. J Clin Epidemiol. 1998 Dec;51(12):1235-41. doi: 10.1016/s0895-4356(98)00131-0. PMID: 10086815.
27. E Lima MT, E Lima JG, de Andrade MF, Bergmann A. Low-level laser therapy in secondary lymphedema after breast cancer: systematic review. Lasers Med Sci. 2014 May;29(3):1289-95. doi: 10.1007/s10103-012-1240-y. Epub 2012 Nov 29. PMID: 23192573.
28. Omar MT, Shaheen AA, Zafar H. A systematic review of the effect of low-level laser therapy in the management of breast cancer-related lymphedema. Support Care Cancer. 2012 Nov;20(11):2977-84. doi: 10.1007/s00520-012-1546-0. Epub 2012 Aug 9. PMID: 22875413.
29. Maher CG, Sherrington C, Herbert RD, Moseley AM, Elkins M. Reliability of the PEDro scale for rating quality of randomized controlled trials. Phys Ther. 2003 Aug;83(8):713-21. PMID: 12882612. (DOI not available)
30. van Tulder M, Furlan A, Bombardier C, Bouter L; Editorial Board of the Cochrane Collaboration Back Review Group. Updated method guidelines for systematic reviews in the cochrane collaboration back review group. Spine (Phila Pa 1976). 2003 Jun 15;28(12):1290-9. doi: 10.1097/01.BRS.0000065484.95996.AF. PMID: 12811274.
31. Sackett DL. Rules of evidence and clinical recommendations on the use of antithrombotic agents. Chest. 1989 Feb;95(2 Suppl):2S-4S. PMID: 2914516. (DOI not available)
32. World Association of Laser Therapy (WALT). Consensus agreement on the design and conduct of clinical studies with low-level laser therapy and light therapy for musculoskeletal pain and disorders. Photomed Laser Surg. 2006 Dec;24(6):761-2. doi: 10.1089/pho.2006.24.761. PMID: 17199479.
33. Lau RW, Cheing GL. Managing postmastectomy lymphedema with low-level laser therapy. Photomed Laser Surg. 2009 Oct;27(5):763-9. doi: 10.1089/pho.2008.2330. PMID: 19878027.
34. Bramlett O, Daysudov I, Odaira T, Rodriguez BP, editors. The Long-Term Effects of Low Level Laser Therapy (LLLT) Combined with Complex Decongestive Therapy (CDT) in the Treatment of Breast Cancer Lymphedema: A Double-Blind, Randomized, Placebo-Controlled Study2014. (DOI not available)
35. Storz MA, Gronwald B, Gottschling S, Schöpe J, Mavrova R, Baum S. Photobiomodulation therapy in breast cancer-related lymphedema: a randomized placebo-controlled trial. Photodermatol Photoimmunol Photomed. 2017 Jan;33(1):32-40. doi: 10.1111/phpp.12284. PMID: 27943450.
36. Baxter GD, Liu L, Tumilty S, Petrich S, Chapple C, Anders JJ; Laser Lymphedema Trial Team. Low level laser therapy for the management of breast cancer-related lymphedema: A randomized controlled feasibility study. Lasers Surg Med. 2018 Sep;50(9):924-932. doi: 10.1002/lsm.22947. Epub 2018 May 30. PMID: 29851090.
37. Kilmartin L, Denham T, Fu MR, Yu G, Kuo TT, Axelrod D, Guth AA. Complementary low-level laser therapy for breast cancer-related lymphedema: a pilot, double-blind, randomized, placebo-controlled study. Lasers Med Sci. 2020 Feb;35(1):95-105. doi: 10.1007/s10103-019-02798-1. Epub 2019 May 11. PMID: 31079232.
38. Mogahed HGH, Badawy MM, Education NMAAJJOAP, Research. Low-Level laser Diode on post modified Radical Mastectomy Lymphedema: a randomized controlled trial ‎. 2020;10(4):105-9.
39. Ridner SH, Poage-Hooper E, Kanar C, Doersam JK, Bond SM, Dietrich MS. A pilot randomized trial evaluating low-level laser therapy as an alternative treatment to manual lymphatic drainage for breast cancer-related lymphedema. Oncol Nurs Forum. 2013 Jul;40(4):383-93. doi: 10.1188/13.ONF.383-393. PMID: 23803270; PMCID: PMC3887507.
40. Martín ML, Hernández MA, Avendaño C, Rodríguez F, Martínez H. Manual lymphatic drainage therapy in patients with breast cancer related lymphoedema. BMC Cancer. 2011 Mar 9;11:94. doi: 10.1186/1471-2407-11-94. PMID: 21392372; PMCID: PMC3065438.
41. de Sire A, Fusco N, Sajjadi E, Lippi L, Cisari C, Invernizzi M. Lymphedema Rehabilitation Using Self-Adaptive Inelastic Compression in Breast Cancer: A Proof-of-Principle Study. 2021;11(4):1901. Doi: 10.3390/app11041901
42. Tumilty S, Munn J, McDonough S, Hurley DA, Basford JR, Baxter GD. Low level laser treatment of tendinopathy: a systematic review with meta-analysis. Photomed Laser Surg. 2010 Feb;28(1):3-16. doi: 10.1089/pho.2008.2470. PMID: 19708800.
43. Law D, McDonough S, Bleakley C, Baxter GD, Tumilty S. Laser acupuncture for treating musculoskeletal pain: a systematic review with meta-analysis. J Acupunct Meridian Stud. 2015 Feb;8(1):2-16. doi: 10.1016/j.jams.2014.06.015. Epub 2014 Jul 10. PMID: 25660439.
44. Sterne JAC, Savović J, Page MJ, Elbers RG, Blencowe NS, Boutron I, Cates CJ, Cheng HY, Corbett MS, Eldridge SM, Emberson JR, Hernán MA, Hopewell S, Hróbjartsson A, Junqueira DR, Jüni P, Kirkham JJ, Lasserson T, Li T, McAleenan A, Reeves BC, Shepperd S, Shrier I, Stewart LA, Tilling K, White IR, Whiting PF, Higgins JPT. RoB 2: a revised tool for assessing risk of bias in randomised trials. BMJ. 2019 Aug 28;366:l4898. doi: 10.1136/bmj.l4898. PMID: 31462531.

- 1. **Cochrane risk-of-bias for randomized trials (RoB 2)**

| **Study** | **D1** | **D2** | **D3** | **D4** | **D5** | **Overall** |
| --- | --- | --- | --- | --- | --- | --- |
| Kilmartin 2019 |  |  |  |  |  |  |
| Mogahed 2020 |  |  |  |  |  |  |
| Baxter 2018 |  |  |  |  |  |  |
| Storz 2017 |  |  |  |  |  |  |
| Ridner 2013 |  |  |  |  |  |  |
| Lau 2010 |  |  |  |  |  |  |
| Bramlett 2014 |  |  |  |  |  |  |
| Omar 2011 |  |  |  |  |  |  |

| D1 | Randomisation process |
| --- | --- |
| D2 | Deviations from the intended interventions |
| D3 | Missing outcome data |
| D4 | Measurement of the outcome |
| D5 | Selection of the reported result |
|  |  |

Low risk

Some concerns

High risk

**Comparisons of lymphedema volume change represented by p value at the end of treatment session.**

| **Included Study** | **p value** (between group) |
| --- | --- |
| Mogahed [2020] | 0.0001 |
| Kilmartin [2019] | 0.118 |
| Baxter [2018] | NR |
| Storz [2017] | 0.13 |
| Bramlett [2014] | 0.095 |
| Ridner [2013] | 0.422 |
| Lau and cheing [2010] | 0.044 |
| Omar [2011] | 0.01 |

Pre and post treatment volume change (ml) values for laser and control group were not mentioned in every RCT hence they are not represented in the table.

NR= not reported

P < 0.05 significant results
